# Gratitude Intervention Evokes Indebtedness: Moderated by Perceived Social Distance

**DOI:** 10.3389/fpsyg.2022.824326

**Published:** 2022-03-16

**Authors:** Wuming He, Junjie Qiu, Yingying Chen, Yufang Zhong

**Affiliations:** ^1^Guangdong Provincial Key Laboratory of Development and Education for Special Needs Children, Zhanjiang, China; ^2^School of Educational Science, Lingnan Normal University, Zhanjiang, China; ^3^Department of Education and Human Potentials Development Special Education Section of Education, National Dong Hwa University, Hualian, Taiwan; ^4^College of Teacher Education, Lingnan Normal University, Zhanjiang, China

**Keywords:** gratitude intervention, indebtedness, perceived social distance, gratitude education, multi-categorical antecedent moderation

## Abstract

Previous study suggests that gratitude intervention evokes indebtedness among people from an interdependent society. This study furtherly hypothesized that perceived social distance moderates the effect of gratitude intervention on felt indebtedness. A total of 275 adolescents were randomly assigned to three gratitude intervention conditions, namely, writing gratitude to significant others, the health of one’s own, or nothing. After completing the writing task, they rated their experienced emotions on ten dimensions, including gratitude and indebtedness. They also reported perceived social distance from surrounding people and other demographical information. Results indicated that participants in the condition of writing about gratitude to significant others felt indebted regardless of perceived social distance, while those in the condition of writing about gratitude to his/her own health and those in the control condition experienced lesser indebtedness as the perceived social distance with others becomes closer. Gratitude increases as perceived social connectedness increases across all conditions. Theoretical and practical implications were discussed.

## Introduction

*Gratitude* is a positive emotion evoked by receiving favors, which promotes relationship quality and prosocial behavior (Emmons and McCullough, 2004; Algoe et al., 2013). *Indebtedness* is a negative emotion experienced when people receive helps from others and feel an obligation to repay the “debts,” which is often undesirable (Greenberg and Westcott, 1983; Fredrickson, 2004; Shen et al., 2011). Both emotions are rooted in social interactions and are interpersonal emotions. They are similar in social nature and sometimes are regarded as the same feelings. For instance, a self-report study found that gratitude and indebtedness are interchangeable to some degree in a lay person’s sense after receiving others’ help (Tesser et al., 1968), that is, people use the pair of gratitude and indebtedness as a synonym to label their experience incurred by others’ help.

However, accumulating studies endeavored to distinguish between gratitude and indebtedness. Gratitude and indebtedness are similar in their social nature but different in valence and motivating drive (Greenberg, 1980). First, gratitude and indebtedness are different in emotional valence, with the former being positive and the latter being negative (Greenberg, 1980; Mayer et al., 1991; Lambert et al., 2009). Second, although both emotions can function as motivating drives to reciprocity, different mechanisms underlie it. Gratitude promotes people’s prosocial tendency, while indebtedness leads to the feeling of obligation to repay the beneficiary’s “debts” to the benefactor (Greenberg and Westcott, 1983). The underlying gratitude is often considered voluntary, whereas indebtedness can make people experience stress (Greenberg, 1980) and they try to avoid it (Shen et al., 2011).

Previous studies have emphasized how gratitude and indebtedness influence behaviors of social proximity (Cong et al., 2017). However, does perceived interpersonal distance shape the feeling of gratitude and indebtedness? Factors influencing emotional responses of gratitude and indebtedness can include the value perceived by the recipient of the outcome of the favor (Tsang, 2007), the cost beard by the benefactor (McCullough and Tsang, 2004), and the nature of the helper’s intentionality (Tsang, 2006; Watkins et al., 2006). However, perceived social proximity as an antecedent received little attention in indebtedness scholarship. Perceived social distance is a self-report measure of perceived distance from others (family members, friends, community, and society). It has been linked to athletic dress style (St-James et al., 2006), online words of mouth (Yang, 2019), disagreement between patients and general practitioners (GP) on the patient’s health status (Schieber et al., 2013), the influence of media on personal risk perception (So and Nabi, 2013), and money priming (He, 2019). Regarding social emotions, previous studies have shown that interpersonal closeness can predict interpersonal forgiveness (Wenzel and Okimoto, 2012). People are more likely to forgive offenders perceived closer to them (Harber and Wenberg, 2005; Karremans et al., 2011). The intention to forgive is suggested to be evoked almost subconsciously and demands relatively little effort in closer relationships (Karremans and Aarts, 2007). Considering these emotions are all contextualized in social relations, it is reasonable to expect similar patterns between them.

Recently, Oishi et al. (2019) found that people are more likely to feel indebtedness in the socially interdependent context, while in the socially independent context, people are less likely to feel indebtedness. According to Oishi et al. (2019), interpersonal connections in an interdependent society are characterized by mutual obligation and in an independent society by mutual liking, which leads to the hypothesis that people from an interdependent society are more likely to feel indebted when gratitude is evoked.

In this study, we extended this line of theorizing by inferring that even in a socially dependent culture, perceived interpersonal closeness would influence individuals’ feeling of indebtedness when gratitude is triggered. Instead of treating interdependency as a demographical factor (Oishi et al., 2019), which qualitatively categorized interdependency into dichotomous conditions, we used a quantitatively measured index of perceived social distance as a proxy of interdependency. Given the social nature of indebtedness, empirical evidence showing the qualitatively categorized high/low social distance influencing indebtedness (Oishi et al., 2019) and the similar pattern of perceived social distance on forgiveness (e.g., Wenzel and Okimoto, 2012), we hypothesized that perceived social distance would moderate the effect of gratitude intervention on the feeling of gratitude and indebtedness. Specifically, we hypothesized that gratitude intervention involving social relations (vs. other situations) would evoke a stronger feeling of gratitude and indebtedness. However, regarding the modulating effect of perceived social distance, we did not make a specific prediction about the directions given the lack of research on the directions of the correlations among gratitude, indebtedness, and perceived social connectedness.

## Materials and Methods

### Participants

A total of 275 adolescents (male: 164) were selected from a secondary school took part in the study, with their ages ranging from 13 to 19 (*M* = 16.53, *SD* = 1.15) years. Before sampling, we first estimated the sample size necessary to achieve a power level of 0.80 for a medium-sized effect of Cohen *d* = 0.20 based on previous research (Oishi et al., 2019). The analysis using G*Power (Faul et al., 2009) suggested a total sample size of 246.

### Manipulation of Gratitude Intervention

We followed the previous study to manipulate the gratitude intervention (Emmons and McCullough, 2004; Oishi et al., 2019). In a writing task, participants were asked to write to express gratitude to their health status (the health condition) or one of the significant others in their life (the SO condition). In the control condition, participants did not complete the writing task.

### Mood Measures

Following previous work by Oishi et al. (2019), participants were asked to complete a 7-point scale indicating their current mood on “happy,” “satisfied,” “pride,” “gratitude,” “appreciation,” “sad,” “anger,” “indebtedness,” “shame,” and “guilt.”

### Perceived Social Distance

Perceived social distance was measured using the Inclusion of Other in Self Scale (Aron et al., 1992), which is a psychologically meaningful and highly reliable measure of subjective interpersonal connectedness (Gächter et al., 2015) and is widely and successfully used in prior studies (Capaldi and Zelenski, 2016; He, 2021). It contains 4 items measuring the perceived social distance from one’s friends, family, community, and society, respectively. Each includes seven pairs of circles with different levels of degree of overlapped areas. Participants can rate their perceived social distance on these seven-point Likert-type scales, with a greater degree of overlap between the pair of circles indicating greater perceived social connectedness. The score on each item was encoded from 1 to 7. A composite score was created by averaging these four items (α = 0.76). A higher score represents a closer perceived social distance.

### Procedure

All participants were randomly assigned to three conditions. After the writing task, they completed the mood measure and reported perceived social distance and other demographical information including gender and age.

## Results

### The Effect of Gratitude Interventions on Emotions

We conducted between-subject ANOVAs to analyze the effect of gratitude intervention on participants’ reported emotions. A marginally significant result has been generated on indebtedness [*F*(2, 272) = 2.58, *p* = 0.077, η^2^ = 0.02], and *post-hoc* comparisons indicated that participants in the SO condition (*M* = 3.37, *SD* = 1.96) felt greater indebtedness than those in the control condition (*M* = 2.73, *SD* = 1.86), *p* = 0.024. Expectedly, participants in the health condition (*M* = 3.12, *SD* = 1.81) felt greater indebtedness than those in the control condition and felt lesser indebtedness than those in the SO condition. However, this pattern was not statistically significant. Significant results also emerged on gratitude and appreciation, with *F*(2, 272) = 9.63, *p* < 0.001, η*^2^* = 0.07 and *F*(2, 272) = 9.73, *p* < 0.001, η*^2^* = 0.06, respectively. Participants in the SO condition (*M* = 5.37, *SD* = 1.80) and the own health condition (*M* = 5.19, *SD* = 1.62) reported greater appreciation than those in the control condition (*M* = 4.22, *SD* = 2.09, both reported *p* < 0.001). Participants in the SO condition (*M* = 5.78, *SD* = 1.62) reported higher gratitude than those in the own health condition (*M* = 5.13, *SD* = 1.82, *p* = 0.013), which was higher than those in the control condition (*M* = 4.58, *SD* = 1.98, *p* = 0.043). More details are presented in Table 1. As the results on appreciation and gratitude showed similar patterns, they were highly correlated, *r*(275) = 0.76, *p* < 0.001, and the word of appreciation and gratitude are synonyms (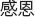 vs. 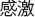) in Chinese context, we will only include the gratitude in the following analysis.

**TABLE 1:** Effects of gratitude interventions on the emotions.

	Gratitude intervention				
	Gratitude to significant other	Gratitude to one’s own health	The control condition	*F*	*p*
Happy	4.24^a^	3.85^a^	4.23^a^	1.66	0.193
Satisfied	3.95^a^	4.04^a^	3.70^a^	0.86	0.424
Pride	3.62^a^	3.55^a^	3.23^a^	1.02	0.361
Gratitude	5.78^a^	5.13^b^	4.58^c^	9.63	<0.001
Appreciation	5.37^a^	5.19^a^	4.22^b^	9.73	<0.001
Sad	2.31^a^	2.59^a^	2.43^a^	0.69	0.504
Anger	1.71^a^	1.95^a^	2.09^a^	1.52	0.221
Indebtedness	3.37^a^	3.12^ab^	2.73^b^	2.58	0.077
Shame	2.93^a^	2.86^a^	2.46^a^	1.90	0.152
Guilt	3.04^a^	3.14^a^	2.69^a^	1.39	0.250

*Means with different subscripts differ at the p = 0.05 level by post-hoc comparisons.*

### Interaction Between Gratitude Interventions and Perceived Social Distance in Terms of Indebtedness

Using simple indicator coding (e.g., “dummy variable”), we employed the PROCESS SPSS application (Hayes, 2013) to construct a multi categorical-continuous interaction model (Preacher and Hayes, 2004; Hayes and Preacher, 2013) to test the moderating effect of gratitude intervention and perceived social distance on indebtedness. Of our interest, the relative conditional effect of the gratitude to health relative to the control condition (reference condition) did not change as the perceived social distance changes (*b* = 0.27, *p* = 0.262). Expectedly, the relative conditional effect of the gratitude to the SO condition compared with the control condition differed by 0.50 units between two participants who differ by one unit in their perceived social distance (*b* = 0.50, *p* = 0.033). The result means that the perceived social distance moderated the relative effect between the significant other condition and the control condition on the degree of indebtedness.

**FIGURE 1:**
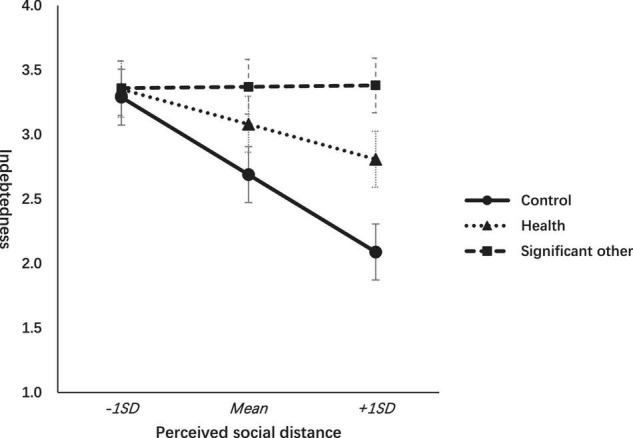
Effects of gratitude interventions on indebtedness across levels of perceived social distance.

For ease of interpretation, the pattern of the results is graphically illustrated in Figure 1.

As shown in Figure 1, participants in the gratitude to significant other conditions (vs. other conditions) experienced greater indebtedness regardless of perceived social distance. Nevertheless, participants in the gratitude to the health condition and the control condition experienced declining indebtedness as the perceived social distance was closer. As the perceived social distance became closer, participants in the control condition and health condition were free from indebtedness to some degree, but the feeling of indebtedness experienced by those in the SO condition remained its strength.

**FIGURE 2:**
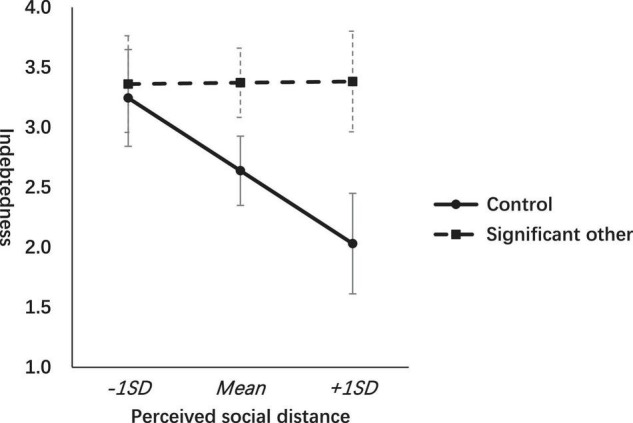
Effects of gratitude interventions (significant other vs. control) on indebtedness across levels of perceived social distance.

To further illustrate this interacting effect, we split the data to focus only on the condition of gratitude to the SO condition and the control condition. We tested the moderation model using Hayes’ PROCESS Macro (Hayes, 2013) with 5,000 bootstrap samples. The perceived indebtedness was entered as the dependent variable with the intervention conditions (gratitude to SO = 1 vs. neutral = 0) as the independent variable and the social distance as the moderator while controlling for age and gender. The results showed that the interaction term between gratitude intervention and perceived social distance was significant (*b* = 0.52, *t* = 2.17, *p* = 0.031). The effect of gratitude intervention (SO vs. control) on indebtedness was significant among participants with closer perceived social distance (*b* = 1.53, *t* = 3.33, *p* = 0.001, 95% CI: 0.62, 2.44) but not significant for those with farther perceived social distance (*b* = 0.12, *t* = 0.30, *p* = 0.765, 95% CI: -0.68, 0.93) (refer to Figure 2).

### Interaction Between Gratitude Interventions and Perceived Social Distance in Terms of Gratitude

**FIGURE 3:**
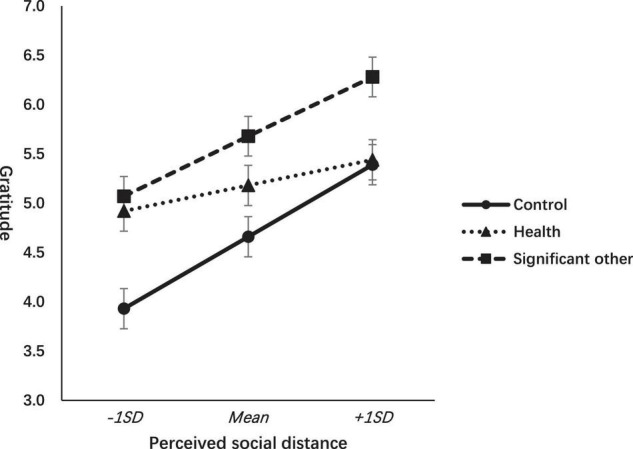
Effects of gratitude interventions on gratitude across levels of perceived social distance.

To test the moderating effect of gratitude intervention and perceived social distance on gratitude, we also employed the PROCESS SPSS application (Hayes, 2013) to construct a multi categorical-continuous interaction model (Preacher and Hayes, 2004; Hayes and Preacher, 2013) on the emotion of gratitude by using indicator coding. The relative conditional effect of the writing gratitude to one’s own health and the writing gratitude to significant others relative to the control condition did not change as the perceived social distance changes by one unit (*b* = -0.37, *p* = 0.101) and (*b* = -0.10, *p* = 0.661), respectively. No interaction effect exists when *F*(2, 269) = 1.55, *p* = 0.214. As the perceived social distance becomes closer, the feeling of gratitude increases. The pattern between the control condition and the SO condition is parallel. The pattern in the health condition seems relatively weak compared with the other two conditions in the illustrated figure, which may be attributed to the writing gratitude object being relatively abstract, but are not related to others. In fact, gratitude is conceptualized as a social emotion. However, the difference in the pattern is not statistically significant (refer to Figure 3).

## Discussion

In this study, we hypothesized that the effect of gratitude intervention on indebtedness is moderated by perceived social distance. The findings confirmed our hypothesis by demonstrating that participants in the condition of writing about gratitude to significant others felt indebted, regardless of perceived social connectedness, while those in the condition of writing about gratitude to his/her own health and those in the control condition experienced lesser indebtedness as the perceived social distance from others was closer.

This study contributes to theoretical questions about when gratitude is likely to be evoked simultaneously with indebtedness. Participants in the SO condition felt greater indebtedness than those in the control condition. This result is consistent with Oishi’s work (Oishi et al., 2019) that participants from an interdependent culture felt indebted when writing gratitude to someone important. In addition, participants in the SO condition experienced the same strength of indebtedness and did not change as the perceived social distance varied from farther to closer. This result suggests that just making people notice the benefit they received from the person they value can evoke indebtedness. The extent of relationship closeness between them is not significant in this circumstance. This result added to the similar previous findings suggest that the feeling of indebtedness that reacts to aid did not vary as a function of helper intention that is benevolent or ulterior (Tsang, 2006). The cue of kindness from others gives rise to the obligation to repay spontaneously. In sum, the findings consolidate the assumption that indebtedness is an interpersonal emotion and serves as the drive of reciprocity norm. Another possible explanation might refer to the threat-to-self-esteem theory of indebtedness, which asserts that, if the benefit is perceived as threatening, then reactions are defensive (Fisher et al., 1982; Fisher, 1983). Additionally, research suggests that aid from a similar and resourceful other person is more self-threatening than the same help from dissimilar other people (Fisher and Nadler, 1974, 1976). Therefore, writing gratitude to someone important may imply relative inferiority and dependency of the participant on the similar and resourceful figure, which, in turn, prompts indebtedness because adolescents in interdependent cultures normally rely on significant others for support and help. Indebtedness further drives people to repay the benefactor or devote themselves to self-help (Greenberg, 1980).

In the control condition, when the participants did not write gratitude and just reported their emotions, participants’ indebtedness is negatively correlated with perceived social distance, with closer perceived social distance relating to lower perceived indebtedness. The equity theory (Fisher, 1983) assumed that people aspire to maintain equity in their interpersonal relationships. If inequity occurs, people will feel discomfortable and are driven to reduce the unpleasant feelings. Therefore, if the beneficiary of help expects to have opportunities to reciprocate in the future, then he/she has less need for immediate reciprocity. For example, when they receive help from friends or members of a closely related group, their desire for immediate reciprocity is lesser (Nadler et al., 1974; Bar-Tal et al., 1977). It is reasonable to expect that, when the perceived connectedness with others increases, people from an interdependent society expect many opportunities for reciprocation. Therefore, with closer perceived social distance, their feeling of indebtedness is generally lower. The drive to immediate reciprocation is not as urgent as those perceived farther social distance. In contrast, those with farther perceived social distance experience a generally higher level of indebtedness. Combinedly, the pattern of results suggests that writing about gratitude to someone significant felt indebted, and the strength of this feeling was independent of perceived social distance. In the conditions of writing about gratitude to one’s health or nothing, indebtedness decreases with closer perceived social distance.

In the condition of writing gratitude to someone important, as the perceived social distance increased, the felt gratitude also strengthened, which means that when expressing gratitude to someone, the feeling of gratitude increases if he/she is perceived to be closer. This finding is consistent with the find-remind-and-bind theory, suggesting that the emotion of gratitude serves a social function of reminding us of current high-quality relations and helping to bind that person more closely (Algoe et al., 2008; Algoe, 2012). It seems that expressing gratitude contributes to both close human connections and feelings of gratitude. This finding for gratitude is also similar to other social emotions, such as forgiveness, which is more likely to be evoked as the perceived social distance decreases (Harber and Wenberg, 2005; Karremans and Aarts, 2007; Karremans et al., 2011).

These findings also add to the literature on the distinction between gratitude and indebtedness (Tsang, 2006; Watkins et al., 2006; Cong et al., 2018) by demonstrating that gratitude and indebtedness can be evoked simultaneously, but their relations to perceived social distance are different. Gratitude serves morality, which is sensitive to the perceived social distance (Hofer et al., 2021). However, indebtedness may not be related to perceived social distance from the benefactor.

This study also provides practical insights into gratitude education, a class of education activities through which the value of gratitude in an interdependent society is presumably transferred to younger adolescents before they become adults. The gratitude education manifests in various forms, including lectures, a footbath for parents, blessing letters to significant others, and the like. All of which are common in expressing gratitude to parents or other significant family members. The practice of gratitude education is the daily life counterpart of gratitude intervention in lab studies.

According to our findings, these activities will evoke not only the feeling of gratitude but also the experience of indebtedness (i.e., in the higher perceived social distance condition). So, gratitude might be intertwined with indebtedness in gratitude education. Gratitude education can produce undesired effects on students. Indebtedness is a negatively valenced emotion, which generates discomfort or anxiety. If the beneficiary believes that the benefactor suffers deeply in helping him, he will feel negative in the gratitude response (Manela, 2016). People reporting grateful feelings also tend to report being somewhat indebted (Watkins, 2014). This might explain why many adolescents in those activities experience negative feelings and display negative behaviors such as crying or grimacing (Netease, 2014; Liu, 2017). For instance, an empirical study suggests that reciprocity of students is not a prosocial behavior but a passive behavior due to pressure from morality; thus, gratitude education does not produce positive emotional experience and social behavior, deviating from the traditional meaning of gratitude in morality building (Pu and Zhu, 2012). Indebtedness subsequently motivates adolescents to behave reciprocally to compensate for the felt inequity in the future. Previous studies suggest that indebtedness can lead to aversive effects on personal wellbeing, such as lowering self-esteem, compromising autonomy (Fisher, 1983; Greenberg and Westcott, 1983), and producing negative evaluations of the benefactor (Greenberg, 1980).

This study has several limitations that should be acknowledged. First, the participants are limited to adolescents. There is a lack of indebtedness research on adolescents cited in constructing our hypotheses and selected adolescents rather than adults as participants. It should be cautious when extending the results to adults. However, previous studies have examined the effect of gratitude intervention on adolescents and have found consistent consequences with adults (Lambert and Veldorale-Brogan, 2013). In terms of indebtedness, about 30% of adolescents (vs. about 40% of adults) reported feeling indebted when explaining why gratitude is not always pleasant (Gulliford and Morgan, 2017), which means that gratitude and indebtedness are also simultaneously evoked in adolescents. In addition, linking the effect of gratitude intervention to the usual practice of “gratitude education” in the eastern culture is one of the primary purposes of this study and practical significance. Therefore, this study can be seen as an exploratory attempt to extend the field of indebtedness research to adolescence. Second, a previous study suggests that the nature of the relationship (communal vs. exchange) may influence benefits recipients’ reactions (Williamson et al., 1996). Nevertheless, the link between perceived social distance and communal exchange relationships is not clear yet. More research is needed to tap into the interaction between perceived social distance and distinctive relationships. Third, the participants write gratitude using a pen and paper. We did not prospectively set up a procedure to count the number of words and the time spent on writing. Previous studies that we followed methodologically did not report the time spent and the word counts (e.g., Oishi et al., 2019). However, they might be potential factors that interfere with participants’ moods. Because people’s life story about significant others is generally more content-rich than one’s health, they may spend more on it. Future studies should take these factors into account. Another concern is related to the design of the experiment. Participants in the SO condition memorized and wrote about their interpersonal interaction with significant others. In contrast, participants in the health/control condition were not in an interactive context. A reference condition with interactive nature may be better than a control condition without doing anything in future studies. Despite these limitations, our study provided insights into the theoretical questions about indebtedness and practical questions about gratitude education.

## Data Availability Statement

The raw data supporting the conclusions of this article will be made available by the authors, without undue reservation.

## Ethics Statement

The studies involving human participants were reviewed and approved by the Department of Psychology at Lingnan Normal University. Written informed consent to participate in this study was provided by the participants’ legal guardian/next of kin.

## Author Contributions

WH and JQ conceived of the study, designed the study, and shared the first authorship for repeated discussion about the design, writing, and arrangement of this manuscript. YZ collected the data. All authors analyzed the data and were involved in writing the manuscript.

## Conflict of Interest

The authors declare that the research was conducted in the absence of any commercial or financial relationships that could be construed as a potential conflict of interest.

## Publisher’s Note

All claims expressed in this article are solely those of the authors and do not necessarily represent those of their affiliated organizations, or those of the publisher, the editors and the reviewers. Any product that may be evaluated in this article, or claim that may be made by its manufacturer, is not guaranteed or endorsed by the publisher.
